# Sigmoid-rectal intussusception in an elderly patient: A case report of an unusual presentation of intestinal obstruction

**DOI:** 10.1016/j.ijscr.2023.109018

**Published:** 2023-11-04

**Authors:** Anis Hasnaoui, Racem Trigui, Mohamed Ben Hassine, Ahmed Haggari, Houda Bellamine

**Affiliations:** a Faculty of Medicine of Tunis, Tunis El Manar University, Department of General Surgery, Menzel Bourguiba Hospital, Rue Djebal Lakhdar, 1006 Tunis, Tunisia; b Department of Pathology, Menzel Bourguiba hospital, 7050 Menzel Bourguiba, Bizerta, Tunisia

**Keywords:** Intussusception, Invagination, Aged, Intestinal obstruction, Colonic neoplasms, Case report

## Abstract

**Introduction:**

Sigmoid-rectal intussusception or invagination is an infrequently documented condition in the adult population, with only a handful of cases reported in the medical literature. The underlying pathological mechanism involves impaired peristalsis, often attributed to a malignant tumor.

**Case presentation:**

A 78-year-old patient, with a history of abdominal pain and lower gastrointestinal bleeding, sought care at our emergency department with evident symptoms indicative of large bowel obstruction. Abdominal examination revealed distension and rectal examination found a mass mimicking an internal rectal prolapse. Subsequently, imaging studies confirmed the diagnosis of sigmoid-rectal intussusception. The patient underwent an emergency open sigmoid resection with Hartman's procedure. The postoperative course was uneventful. Anatomopathological analysis revealed the presence of stage I adenocarcinoma. A restoration of digestive continuity was scheduled six months later. One-year follow-up assessments showed no indications of local recurrence or distant metastasis.

**Discussion:**

Sigmoid rectal intussusception stands as a unique and infrequently reported medical entity. The absence of distinct clinical symptoms often renders diagnosis a challenging task, with confirmation typically relying on radiological findings. In contrast to the non-surgical approaches employed in pediatric cases, intussusception in adults necessitates surgical intervention due to its predominantly malignant underpinnings.

**Conclusion:**

While sigmoid-rectal intussusception is an exceedingly rare occurrence, its manifestation with a multitude of non-specific symptoms can complicate clinical recognition. Nevertheless, it should be duly considered as a potential etiological factor in cases of large bowel obstruction, particularly when suggestive signs are found on rectal examination.

## Highlights

• Intussusception is an exceptionally rare occurrence in adults.
• Sigmoid-rectal intussusception is the least common subtype of intestinal intussusception.
• The majority of adult colonic intussusceptions are of malignant origin.
• Sigmoid-rectal intussusception should be considered as a potential cause of large bowel obstruction, warranting a systematic digital rectal examination.
• A pre-resection reduction maneuver is mandatory in this intussusception subtype in order to preserve the rectum.

## 1 Introduction

Sigmoid-rectal intussusception or invagination refers to a clinical scenario in which a portion of the sigmoid colon, known as the “intussusceptum”, telescopes into the lumen of the adjacent rectal segment, termed the “intussuscipiens”. While the precise mechanisms underlying this condition remain unclear, advanced theories center around the correlation between impaired peristalsis and bowel lesions. Although relatively common in pediatric cases, it is an exceptionally rare occurrence in adults, constituting 1–5 % of all cases of bowel obstruction [1,2]. Colorectal intussusception is a serious condition, given its potential to result in bowel obstruction and ischemia. We present a case of sigmoid-rectal intussusception in an elderly patient, revealed by large bowel obstruction, and evoked on digital rectal examination. This case report adheres to the SCARE Criteria [3].

## 2 Case presentation

A 78-year-old patient, with no medical or surgical history, was admitted to our surgery ward due to symptoms suggestive of a large bowel obstruction that had been progressing over the past 24 h. These symptoms included abdominal pain, important abdominal distension, and complete incapacity to pass stool. Over the course of the last two years, the patient had recurrently experienced pain in the left iliac fossa, often accompanied by constipation and hematochezia. As these symptoms resolved spontaneously, the patient did not seek medical attention.

**Fig. 1:**
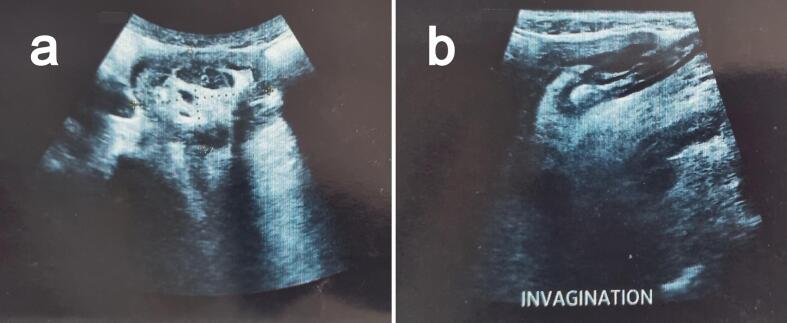
Ultrasonography findings. (a) Transverse view showing the target sign. (b) Longitudinal view showing the pseudo-kidney sign.

On examination, the patient was conscious and had a blood pressure of 120 / 70 mmHg, a pulse rate of 95 beats per minute, and a respiratory rate of 20 breaths per minute. The abdomen was distended, without signs of abdominal masses or incarcerated hernias. The rectal examination was positive for blood and revealed a circular, painless, prolapsed mass engaged in the lower rectum, 5 to 6 cm from the anal canal. While simulating defecation, the mass became circumferential. Urgent complete blood tests showed anemia with a hemoglobin level of 9 g/dl, and normal white blood cells and platelet counts. No renal function impairment or electrolyte disorders were found. An emergency abdominal US (Fig. 1) and CT scan were performed, confirming the diagnosis of sigmoid-rectal intussusception with distension of the descendant colon without signs of bowel ischemia or pre-perforation.

**Fig. 2:**
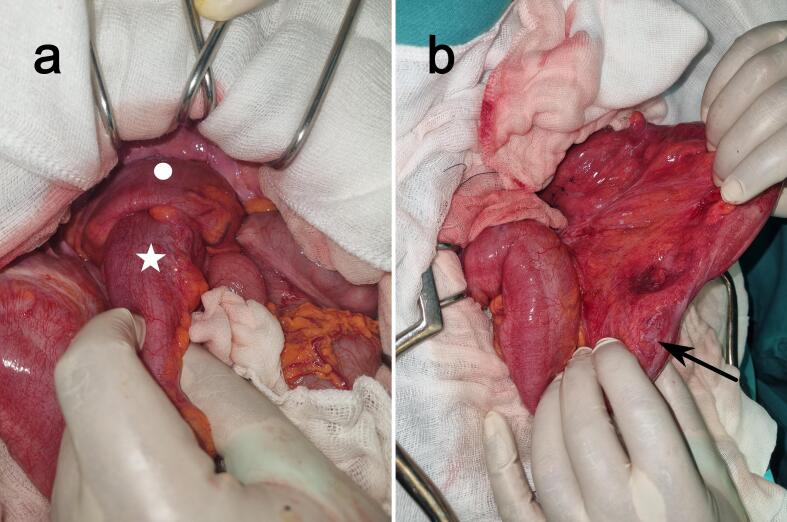
Intraoperative view. (a) Sigmoid-rectal intussusception with the sigmoid indicated by a white star and the high rectum by the white circle. (b) Intraoperative view after the reduction of the intussusception. The tumor location is indicated by a black arrow.

**Fig. 3:**
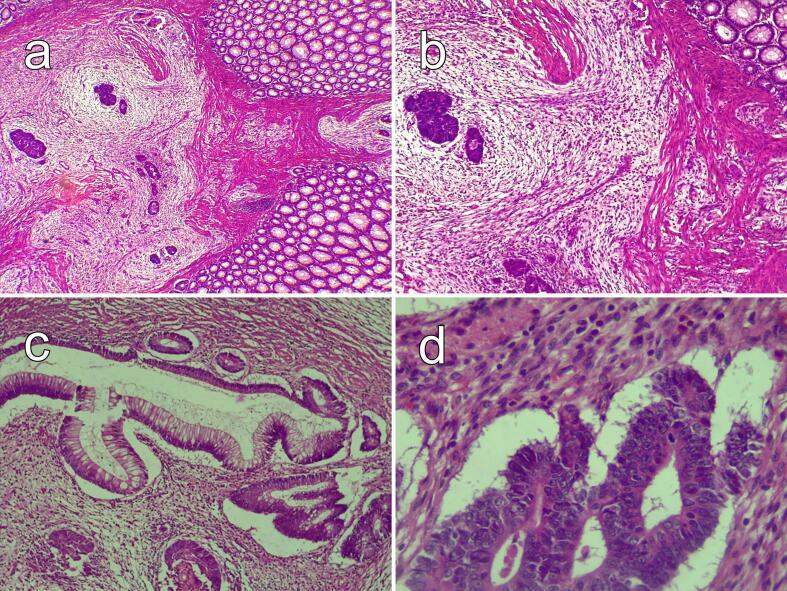
Histopathology findings of the sigmoid tumor. (a) and (b) Infiltration of the colonic wall by glandular and cribriform tumor structures, accompanied by a moderately inflamed loose fibrous stroma, H&E ×40 and ×100 respectively. (c) Infiltration of the colonic wall by glandular tumor structures, H&E ×100. (d) The tumoral cribriform structures are lined with tall cylindrical cells, possessing irregularly stratified nuclei and basophilic cytoplasm with very low mucosecretory activity, H&E ×400.

After a short resuscitation, the patient underwent an urgent laparotomy. Intraoperatively, we discovered a long sigmoid-rectal intussusception (Fig. 2a). The surgeon decided to reduce the intussusception to preserve the rectum, especially with the absence of ischemia or pre-perforation in preoperative images. The intussusception was reduced by adjoining perineal and transabdominal approaches. There was a small (3 to 4 cm) palpable tumor in the sigmoid (Fig. 2b). A carcinologic sigmoidectomy was performed, associated with a Hartman's colostomy. The post-operative course was uneventful. A complete colonoscopy and tumor markers were performed and were within normal limits. The anatomopathological examination revealed a stage I colic adenocarcinoma (Fig. 3). After a multidisciplinary meeting, our decision was not to perform adjuvant chemotherapy. The patient was scheduled for a restoration of digestive continuity six months later and the one-year follow-up did not reveal local recurrence or distant metastasis.

## 3 Discussion

While it is a routine emergency in pediatric practice, adult intussusception is a rare diagnosis that represents 5–10 % of all intussusceptions combined [1], and 1–5 % of all bowel obstruction causes [1,2]. The exact mechanism responsible for adult intussusception is still unknown to this day, but the most plausible theory involves peristalsis impairment, thus explaining cases without leading causes [4]. Risk factors associated with intussusception are yet to be determined, but abdominal surgery is a considerable factor, as invagination often occurs at the junction of moving and fixed bowel segments. Other papers have suggested that any bowel wall lesion or irritant that disrupts peristaltic bowel activity can initiate the invagination process [5]. The etiologies for sigmoid rectal intussusception are varied, ranging from malignant tumors and benign polyps to idiopathic invaginations. According to a literature survey, up to 66 % of colonic intussusceptions are of malignant origin [6]. In our case, the presence of a sigmoid tumor was a promoting factor that led to the sigmoid-rectal intussusception and subsequently the bowel obstruction and prevented spontaneous resolution of the invagination.

Adult intussusception can be classified into four subtypes based on the location of the proximal and distal segments. Among these, sigmoid rectal intussusception stands out as the less frequently encountered subtype, with only a handful of cases documented in comprehensive literature reviews [7,8]. The major challenge facing young surgeons in similar cases is suspecting sigmoid-rectal intussusception as a cause of large bowel obstruction. Even with a complete history intake and thorough physical examination, recognizing this unique entity requires a high index of clinical suspicion. Previous case series and literature reviews consistently underscore that the clinical presentation and duration of symptoms in cases of sigmoid-rectal intussusception are variable and often lacking in specificity. When an organic cause underlies the condition, it typically manifests as a large bowel obstruction, characterized by symptoms such as severe abdominal pain, pronounced abdominal distension, and a complete inability to pass stool. Other non-specific signs can also be found, like gastrointestinal bleeding and diarrhea [1,5,7,8]. Unlike our case where our patient presented with bowel obstruction, most of the patients in the reported literature consulted for chronic symptoms evolving for more than 14 days [7].

Typically, an abdominal physical examination does not reveal specific signs. In a few reported cases, a mass was found protruding throughout the rectum, thus mimicking complete rectal prolapse [[9], [10], [11]]. We believe that one of the features that make this case interesting is the palpable intussusception on rectal examination, which highlights the importance of a complete physical examination to guide the diagnosis. Although disturbed by gas interposition, abdominal ultrasound is a helpful imaging technique if performed by experienced hands in the adult population. Basic signs of intussusception include target and doughnut signs on the transverse view and pseudo-kidney signs on the longitudinal view [12]. These signs were depicted in our case. Abdominal CT is a highly effective imaging method for confirming diagnosis. According to the published case series, abdominal CT scans reached a diagnostic accuracy of 73 %–100 % [13,14].

As for the management of this condition, barium enema is the first line of treatment in pediatric cases. Engaging in this practice with adults is not advised due to heightened concerns surrounding the prevalent malignant etiology associated with adult intussusception [15]. Surgery is the mainstay of treatment, and many important factors affect the surgical approach, such as the presence of a malignant tumor, the location and extension of the intussusception, and the presence of bowel ischemia [16]. The reduction of the intussuscepted bowel gave rise to various concerns regarding postoperative morbidity and long-term survival. Since 2019, caution has been advised against pre-resection reduction due to the theoretical risks of intraluminal seeding and venous embolisms [17]. In our case, we executed a reduction maneuver with the aim of preserving the rectum, particularly given the tumor's location in the sigmoid and the absence of signs of ischemia or pre-perforation. Resection with primary anastomosis or Hartman's procedure can both be performed safely depending on the surgeon's level of expertise, the presence of factors of anastomotic leak, and intraoperative field contamination [18]. In our case, the surgeon opted for an open resection with Hartman's procedure.

## 4 Conclusion

Sigmoid-rectal intussusception is an uncommon occurrence in adults. Due to its non-specific symptomatology, arriving at a diagnosis can pose a challenge. Nevertheless, it should consistently be considered as a potential cause of large bowel obstruction, warranting a systematic digital rectal examination. Employing advanced imaging techniques enables a precise and timely diagnosis. While surgery constitutes the fundamental approach to treatment, it is imperative to carefully weigh various critical factors before determining the course of action for the patient.

## Consent for publication

Written informed consent was obtained from the patient for publication and any accompanying images. A copy of the written consent is available for review by the Editor-in-Chief of this journal on request.

## Ethical approval

Ethical approval was deemed unnecessary by our institutional ethical committee, as the paper is reporting a single case that emerged during normal practice.

## Funding

Nothing to declare.

## Author contribution

Anis Hasnaoui: Conceptualization, Writing-Reviewing and Editing. Racem Trigui: writing-Original draft preparation. Mohamed Ben Hassine: Data curation. Ahmed Haggari: Writing-Reviewing. Houda Bellamine: Data curation. All authors read and approved the final manuscript.

## Guarantor

Anis Hasnaoui

## Research registration number

Not applicable.

## Conflict of interest statement

Nothing to declare.
